# The Role of the pH in the Impregnation of Spherical Mesoporous Silica Particles with L-Arginine Aqueous Solutions

**DOI:** 10.3390/ijms222413403

**Published:** 2021-12-14

**Authors:** Sara Saber Younes Mohamed, Sonia Martinez, Mauro Banchero, Luigi Manna, Silvia Ronchetti, Barbara Onida

**Affiliations:** Department of Applied Science and Technology, Politecnico di Torino, Corso Duca degli Abruzzi 24, 10129 Torino, Italy; sara.mohamed@polito.it (S.S.Y.M.); martinezsonia.sm95@gmail.com (S.M.); mauro.banchero@polito.it (M.B.); Luigi.manna@polito.it (L.M.); silvia.ronchetti@polito.it (S.R.)

**Keywords:** spherical mesoporous silica, arginine, wet impregnation, degradation

## Abstract

In the context of the development of carriers for amino acids delivery, Spherical Mesoporous Silica Particles (SMSP), characterized by particles size ranging from 0.15 µm to 0.80 µm and average pore diameter of 2.4 nm, were synthesised and loaded with L-arginine (ARG), a basic amino acid involved in several physiological processes. The loading was performed using water as a solvent through the wet impregnation method (with a final arginine content of 9.1% *w*/*w*). The material was characterized before and after impregnation by means of X-Ray Diffraction (XRD), nitrogen sorption analysis, Field Emission Scanning Electron Microscopy (FESEM) and Fourier Transform Infrared (FT-IR) spectroscopy. SMSP are shown to suffer degradation upon impregnation, which dramatically affects their porosity. To elucidate the role of the pH of the ARG impregnating solution (originally set at pH ≈ 11) on SMSP degradation, the loading was performed under different pH conditions (5 and 9) keeping constant the ARG concentration. The impregnation performed with acidic solution did not modify the carrier. All samples displayed ARG in amorphous form: zwitterionic species were present in SMSP impregnated at basic pH whereas positive protonated species in that impregnated at acidic pH.

## 1. Introduction

In the last decades, the adsorption of amino acids on the surface of several materials has gained great attention since it is considered of great importance for many applications, including food industry, biodegradable plastic manufacturing, agrochemical, pharmaceutical production, and solid-phase peptide synthesis [1,2,3,4]. Among the different amino acids, L-arginine (ARG) is considered one of the most versatile ones [5]. ARG is the basic amino acid with the highest isoelectric point, which is equal to 10.76 [6]. It presents an amino group (-NH_2_), a carboxylic acid group (-COOH) and a guanidine group (H_2_NC(=NH)NH_2_) in its side chain, which makes it highly hydrophilic [5,7,8]. When ARG is solubilized, these functional groups can be protonated (-NH_3_^+^) or deprotonated (COO^−^) and ARG may exist in the cationic, zwitterionic or anionic form [2,9], which depends on the pH value of the medium. ARG is classified as a semi-essential or conditionally essential amino acid, and it is involved in several physiological processes such as the biosynthesis of essential biomolecules, tissue reparation, cellular regeneration and wound healing. In addition, exogenous administration of arginine seems to have beneficial therapeutic effects including the reduction in the risk of cardiovascular diseases, remission of alopecia, improvement of immunity, enhancement of wound healing and improvement of the vascular endothelial functions [5,7,10,11,12]. However, the administration of ARG presents some limitations. On one hand, oral administration is restricted by the fact that ARG degrades in the intestine tract, so resulting in low bioavailability [11,13]. On the other hand, local application could enhance the treatment efficiency, but this is limited by low cutaneous penetration of arginine due to its hydrophilicity [10]. To overcome all these limitations, researchers suggested the use of nano-systems based on lipids [10], polymers [14], and inorganic materials for the delivery of ARG [13].

Among the different drug delivery systems, mesoporous silica particles have been widely explored due to their unique properties and biocompatibility. These materials are characterized by high specific surface area and pore volume, tuneable pore size, possibility of versatile surface functionalization and high loading capacity of active agents, which may range from small molecules such as drugs and amino acids to bulkier ones like proteins and vaccines [15]. Moreover, mesoporous silica particles are rich in silanol groups on their surface, which could interact with the functional groups of amino acids [2,9] and make these materials suitable carriers for ARG. In addition, the synergy between ARG and mesoporous silica is particularly appealing in the prospect of wound healing applications. The drug topical administration could be combined with the possibility for the carrier to be a source of silicic acid. It was, in fact, proven that the intercellular release of silicic acid from silica nanoparticles may promote in vitro wound healing [16].

Numerous researchers have investigated the adsorption of amino acids on mesoporous silica particles [3,16,17,18], but only few studies are available as far as the adsorption of ARG is concerned. Gao et al. studied the adsorption of ARG on SBA-15 from aqueous solutions at different pHs to understand the main interaction between ARG and the silica surface. The results of their study suggested that the adsorption of ARG on SBA-15 was mainly driven by electrostatic interactions and the adsorption was favoured at high pH rather than low pH, which reached its maximum at pH 10 [2]. Solanki et al. reported the encapsulation of different amounts of ARG into MCM-48 by incipient wetness impregnation and investigated its release behaviour in simulated body fluids under different pH conditions. The results obtained at pH 1.2 showed that, in acidic conditions, the release of ARG was slow while at 7.4 the release was fast. The authors suggested that such behaviour could be ascribed to the different electrostatic interactions arising from the different ionization state of ARG in the aqueous solution [13].

Even though the above-mentioned papers have investigated the possibility of loading ARG on different silica supports, no specific studies regarding the stability of these carriers towards the loading of ARG from aqueous solutions can be found in the literature. This point should deserve particular attention since it is known that silica based materials could dissolve under basic pH conditions [19,20] and ARG solutions are alkaline (pH about 11) [21], which could compromise the pore structure of the support after the drug impregnation. The present paper is focused on the synthesis and characterization of spherical mesoporous silica particles (SMSP) and their impregnation with aqueous ARG solutions at different pHs. The main aim was to investigate at what extent the different pH of the impregnating solutions could affect the structure of the carrier.

## 2. Results and Discussion

This section discusses the characterization results of the mesoporous silica as such (SMSP) and the samples impregnated with aqueous ARG solutions at different pHs. The impregnated samples were named as ARG-x@SMSP, where x indicates the pH value of the ARG/H_2_O impregnating solution.

### 2.1. Characterization of SMSP

The characterization of the unimpregnated material by means of nitrogen sorption analysis and Field Emission Scanning Electron Microscope (FESEM) is discussed hereafter.

**Figure 1:**
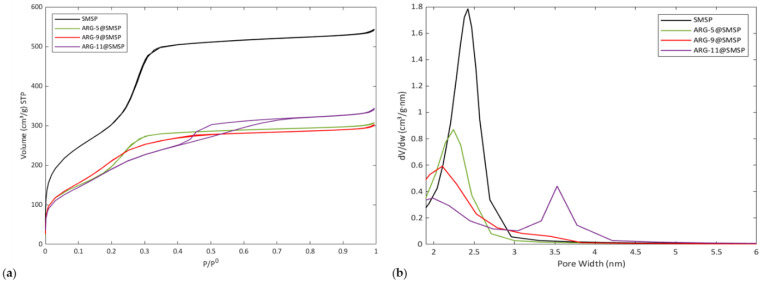
(**a**) Nitrogen adsorption–desorption isotherms and (**b**) pore size distribution of the SMSP, ARG-5@SMSP, ARG-9@SMSP, and ARG-11@SMSP.

Figure 1 shows the nitrogen adsorption–desorption isotherms (section a) and pore size distribution (section b) of SMSP ARG-5@SMSP, ARG-9@SMSP, and ARG-11@SMSP. SMSP presents a type IV isotherm (Figure 1a, black lines), according to IUPAC classification, without any hysteresis loop and shows capillary condensation at relative pressures P/P^0^ between 0.2 and 0.3, so suggesting the presence of uniform mesopores. The pore size distribution (Figure 1b, black line) is unimodal and narrow with an average pore size of 2.4 nm. The values of SSA_BET_ and V_p_ are 1143 m^2^/g and 0.82 cm^3^/g, respectively.

**Figure 2:**
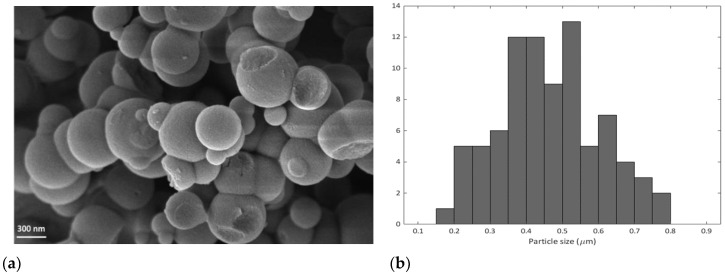
(**a**) FESEM image and (**b**) particle size distribution of SMSP.

Figure 2 reports the FESEM image of SMSP and the calculated particle size distribution. The material appears composed of spheres with a wide particle size distribution ranging from 0.15 µm to 0.80 µm. The wide distribution of the size of the particles could be ascribed to the rapid precipitation and aggregation of the particles during the synthesis process [22].

### 2.2. Characterization of ARG-11@SMSP

The isotherm of ARG-11@SMSP (Figure 1a, violet lines) presents a type H2 hysteresis loop at P/P^0^ above 0.4, which is characteristic of materials with disordered porosity having ink-bottle pores. A remarkable change is also observed in the pore size distribution (Figure 1b, violet line) when compared to SMPS as such: after impregnation the original family of pores with a diameter of 2.4 nm dramatically decreased in volume and its pore size shifted to 2.0 nm, Moreover, a new family of larger pore with diameter of about 3.6 nm appeared. The above-cited changes clearly reveal that, as far as the ARG-11@SMSP is concerned, the exposure to the pH 11 ARG solution during the impregnation process had a significant effect on the textural properties of the carrier. A similar effect was previously observed for different silica-based materials after treatment with basic solutions or after immersion in aqueous media [23,24,25,26]. According to the authors, the variation of porosity was caused by dissolution of the silica walls between the pores, which lead to the formation of larger pores and the generation of soluble silica species that redeposited on the material so resulting in a decrease of the diameter of the pores [23,24,25,26].

Our results suggest that a similar phenomenon could have occurred during the impregnation step since the ARG solution was alkaline, as known by Ninni et al. [21]. During the ARG loading, a gradual dissolution of the silica walls may have occurred so leading to the opening of the interconnections between the pores, which resulted in the formation of new pores with an average diameter of 3.6 nm. Moreover, the decrease of both the volume and the size of the original pores of the unimpregnated SMSP (2.4 nm) may be ascribed to the redeposition of soluble silica species on the pore surface and at the pore mouth. However, the pore size reduction as well as the decrease of the SSA_BET_ and pore volume (Table 1) may partially be due also to the presence of ARG molecules on the silica surface.

In order to confirm that the change in the textural properties was due to the exposure to the alkaline pH during the impregnation, pure SMSP was simply treated with both water (pH 7) and a basic solution (NH_4_OH solution pH 11) with the same procedure used for ARG impregnation, and nitrogen adsorption–desorption analysis on both samples were performed. The isotherm of the sample treated with water (SMSP-H_2_O) (Figure S1) is type IV without hysteresis as observed for that of SMSP. On the other hand, a significant change in the isotherm of the sample treated with the basic solution (i.e., SMSP-NH_4_OH, Figure S1) with the occurrence of a type H2 hysteresis is observed. These results strongly indicate that degradation of SMSP depends on the pH of the impregnated solutions.

**Figure 3:**
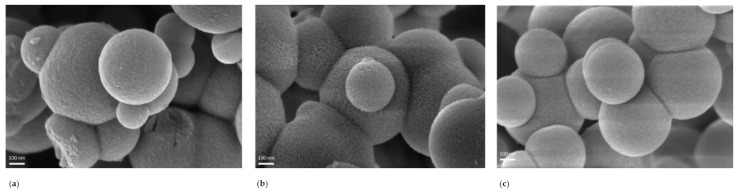
FESEM image (**a**) SMSP, (**b**) ARG-11@SMSP, and (**c**) ARG-5@SMSP.

Figure 3 shows the FESEM images of SMSP, ARG-11@SMSP, and ARG-5@SMSP. As far as sample ARG-11@SMSPs (Figure 3b) is concerned, the morphology does not change when compared to the as-synthesized material, i.e., the particles are still spherical, but a rough surface is observed, which is not present in the unimpregnated SMSP. The rough surface could be ascribed to the degradation of SMSP due to the partial dissolution of silica when contacted with the basic solution during the loading process. These results are consistent with those obtained by the nitrogen sorption analysis, suggesting that SMSP are unstable to the impregnation process and suffer degradation.

**Figure 4:**
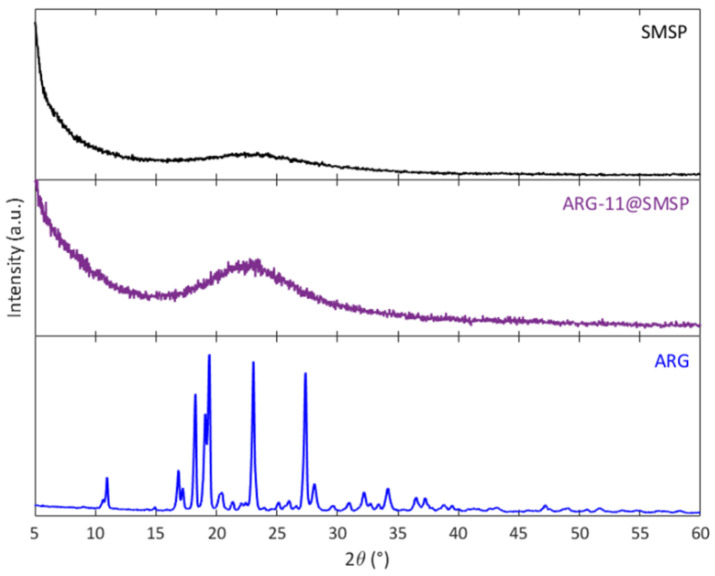
XRD patterns of SMSP, ARG-11@SMSP and pure ARG.

Figure 4 compares the X-ray diffraction (XRD) patterns of SMSP, ARG-11@SMSP, and pure ARG. The XRD pattern of pure ARG reveals well-defined peaks of the crystalline phase. The XRD patterns of SMSP and ARG-11@SMSP are typical of amorphous silica and no peaks due to crystalline ARG are observed for the ARG-11@SMSP samples, revealing the presence of ARG in the amorphous form.

**Figure 5:**
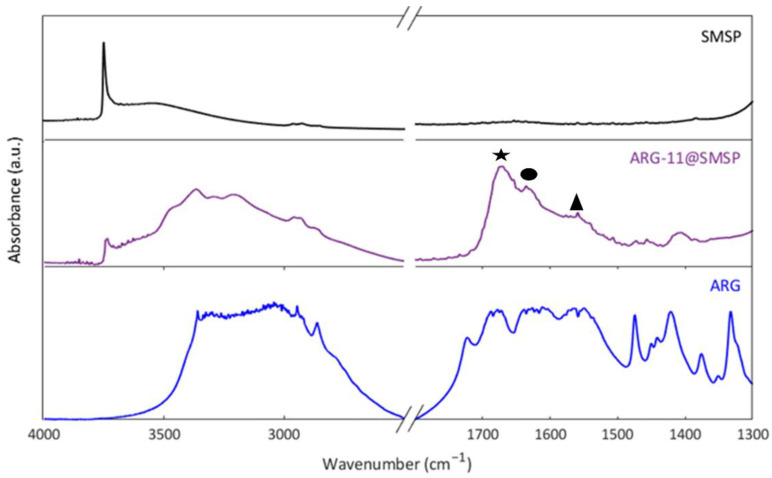
FT-IR spectra of SMSP, ARG-11@SMSP and ARG outgassed at room temperature.

Figure 5 shows the Fourier Transform Infrared (FT-IR) spectra of SMSP and ARG-11@SMSP (the spectrum of pure ARG is also reported for comparison). The spectrum of SMSP is typical of amorphous silica, with the narrow band at 3746 cm^−1^ due to the isolated silanols and a broad absorption at about 3530 cm^−1^ due to H-bonded silanols [27]. In the spectrum of ARG-11@SMSP a reduction of the intensity of the peak associated to isolated silanols is observed. Moreover, a new broad absorption appears in the range 3500–2500 cm^−1^, to which several narrower bands are superimposed. The lower intensity of the peak due to isolated silanols suggests that in ARG-11@SMSP these are perturbed, probably by the H-bonding interaction with the ARG molecules [28], so forming the new broad absorption below 3500 cm^−1^. The narrower bands in the range 3500–3100 cm^−1^ and below 3000 cm^−1^ are ascribed to the NH stretching and CH stretching modes of ARG molecules, respectively. Other several bands due to adsorbed ARG molecules are observed in the 1700–1300 cm^−1^ range. In particular, the two bands at 1670 cm^−1^ (labelled with the star in Figure 5) and at 1630 cm^−1^ (labelled with the circle in Figure 5) correspond to the antisymmetric and symmetric stretching of the guanidine group, respectively [29]. A shoulder is observed at about 1560 cm^−1^ (labelled with the triangle in Figure 5), which can be assigned to the antisymmetric stretching of COO^−^ carboxylate groups [30]. The above results suggest the presence of the zwitterionic form of ARG in the ARG-11@SMSP sample, which is in accordance with the ionization state of the amino acid in the impregnation solution at a pH value of about 11 [2].

### 2.3. Characterization of ARG-5@SMSP and ARG-9@SMSP

In order to confirm the role of the pH of the impregnating solution in the degradation of the SMSP, the loading process was carried out at different pH values while the ARG concentration of the solution was kept constant.

Figure 1 reports the nitrogen adsorption–desorption isotherm (section a) and pore size distribution (section b) for all samples. Upon decreasing the pH of the impregnation solution, the shape of the isotherms changes and the hysteresis loop disappears. The isotherm of the sample impregnated at acidic pH, which is ARG-5@SMSP (Figure 1a, green lines), is similar to that of SMSP as such. The values of SSA_BET_ and pore volume of ARG-5@SMSP are 792 m^2^/g and 0.46 cm^3^/g, respectively, i.e., lower than those measured for the silica before impregnation (Table 1), and this is ascribed to the presence of the loaded ARG molecules.

In addition, the pore size distribution changed upon lowering the pH of the impregnating solution. The family of pores with diameter 3.6 nm almost disappeared for ARG-9@SMSP (Figure 1b, red line) and the pore size distribution of ARG-5@SMSP (Figure 1b, green line) is unimodal with an average pore size of 2.2 nm. As previously discussed for the SSA_BET_ and pore volume values, the lower average pore size of the ARG-5@SMSP sample (2.2 nm) with respect to the SMSP as such (2.4 nm) can be ascribed to the presence of ARG molecules inside the mesopores or at the pores mouth.

All results confirm that the degradation of SMSP is due to the pH of the ARG solution employed for the impregnation and is not to be related to the interaction of the silica with ARG. In particular, this degradation, which affects the porosity, occurs at pH values between 9 and 11. The SMSP are not inert towards the ARG loading process when water is used as a solvent, even though a small amount of solution is used as in the present case, i.e., about 2 mL of impregnating solution for 200 mg of silica support (Section 3.3).

With respect to the previously discussed results, the impregnation at pH 5 allows the integrity of the SMSP carrier to be maintained. Figure 3c shows the FESEM micrograph ARG-5@SMSP. In contrast to what observed for the ARG-11@SMSP sample (Figure 3b), no morphology change or roughness of the particle surface can be observed for ARG-5@SMSP (Figure 3c) when compared to the as-synthesised material. This suggests that the impregnation process performed using an acidic ARG solution does not cause any degradation of SMSP, in agreement with the results obtained from nitrogen sorption analysis (Figure 1) and data reported in literature about the stability of mesoporous silica in acidic solutions [19,20].

**Figure 6:**
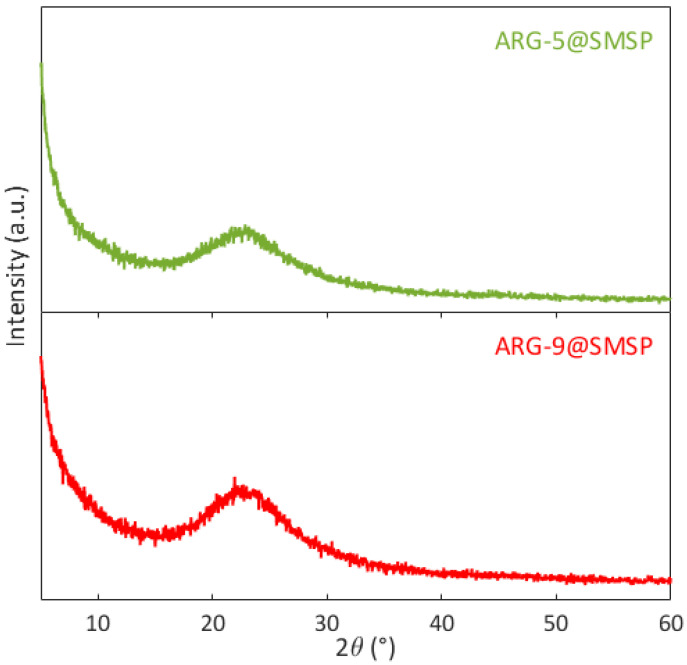
XRD patterns of ARG-5@SMSP and ARG-9@SMSP.

The XRD patterns of ARG-5@SMSP and ARG-9@SMSP are compared in Figure 6. In both patterns the peaks of crystalline arginine are not observed, which suggests the presence of arginine in the amorphous form in both ARG-5@SMSP and ARG-9@SMSP, as observed for ARG-11@SMSP.

**Figure 7:**
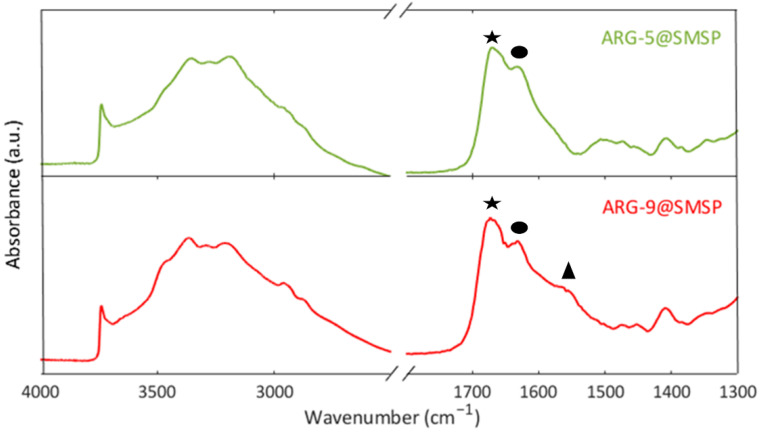
FT-IR spectra of ARG-5@SMSP and ARG-9@SMSP outgassed at room temperature.

Figure 7 shows the FTIR spectra of ARG-5@SMSP and ARG-9@SMSP. In the spectra of both ARG-5@SMSP and ARG-9@SMSP the low intensity of the band due to isolated silanols at 3746 cm^−1^ and the broad absorption between 3500 cm^−1^ and 2500 cm^−1^ reveal the occurrence of H-bonding interactions between the silica surface and the ARG molecules. For the ARG-9@SMSP sample the spectrum in the 1700–1300 cm^−1^ range is very similar to the one reported for ARG-11@SMSP (Figure 5), indicating the presence of the zwitterionic form of arginine. Instead, in the spectrum of ARG-5@SMSP the antisymmetric stretching of COO^−^ at about 1560 cm^−1^ disappeared, revealing the protonation of the carboxylate groups, as expected for ARG molecules in solution at pH between 4 and 8 [2].

A preliminary desorption test was carried out on sample ARG-5@SMSP for which no evidence of degradations was observed. The results showed a complete release of ARG in water from the sample within 10 min, so confirming the possibility to use the SMSP carrier for arginine delivery.

## 3. Materials and Methods

### 3.1. Materials

Ammonium hydroxide solution (ACS reagent, 28.0–30.0% NH_3_ basis), hexadecyltrimethylammonium bromide (CTAB, BioXtra, ≥99%), tetraethyl orthosilicate (TEOS, 99.999% trace metals basis), L-arginine (>99.5%), hydrochloric acid (ACS reagent, 37%), acetic acid (≥99.8%), and ninhydrin were purchased from Sigma-Aldrich (St. Louis, MO, USA). Anhydrous sodium acetate (>99.99%) was provided by Fluka (Buchs, Switzerland). Anhydrous absolute ethanol was purchased from Carlo Erba (Val-de-Reuil, France) and water (LC-MS grade) was provided by Merck (Billerica, MA, USA).

### 3.2. The Synthesis of SMSP

The SMSP were synthesized following the procedure reported by Ambati et al. [23], except for the employed amount of reagents, which was doubled in order to obtain a larger quantity of material. An amount of 2.2 g of CTAB were dissolved in a mixture of 41.8 g of H_2_O, 53.6 g of C_2_H_5_OH and 13.8 g of aqueous ammonia. The solution was stirred for 30 min, then 4.2 g of TEOS were added dropwise. The mixture was further stirred for 2 h at room temperature; the resulting white solid was then filtered, washed with water, and dried for one night. The dried material was calcined in air at 500 °C for 4 h in a muffle furnace to remove the template. The molar ratios of the reactants were 1 TEOS: 0.3 CTAB: 11 NH_3_: 58 C_2_H_5_OH: 144 H_2_O. From the above-described synthesis an amount of 1.1 g of SMSP was eventually obtained.

### 3.3. Loading and Desorption of ARG

The loading of ARG was performed using water as a solvent by the wet impregnation method and according to the procedure reported by Solanki et al. [13]. The volume of solution employed for loading is about 10 times larger than that of the SMSP pore volume, therefore this method has to be considered a wet impregnation and not an incipient wetness impregnation method, where the volume of solution has to correspond to the pore volume of the material. Briefly, 200 mg of SMSP were impregnated with 2 mL of ARG/H_2_O solution (10 mg/mL) so that the nominal ARG content was about 10% *w/w*. The pH of the solution was determined using pH-sensitive litmus paper and was about 11. The sample was then dried at 60 °C in an oven till complete evaporation of water. The obtained material was named as ARG-11@SMSP. In order to check if the effective ARG amount was comparable to the nominal content, thermogravimetric analyses in air were performed on the SMSP before impregnation and on the ARG-11@SMSP sample, using a Linseis STA PT 1600 (TGA-DSC) instrument. Temperature was raised from 30 °C to 800 °C at a heating rate of 10 °C/min in air. The ARG content in the ARG-11@SMSP sample was evaluated from the weight loss between 200 °C and 800 °C and by subtracting the weight loss measured in the same temperature range for the unimpregnated SMSP (Figure S2). The measured ARG content in the ARG-11@SMSP sample resulted to be about 9.1% *w*/*w*, which can be considered in good agreement with the nominal content (10% *w*/*w*).

The loading of ARG at different pH values was carried out with the same procedure previously described. The pH of the ARG/H_2_O impregnating solutions was adjusted to the desired value (5 and 9) with the addition of HCl (1% *w*/*w*). A volume of about 2 mL of the prepared solutions was added to SMSP powder. The obtained materials were named as ARG-x@SMSP, where x indicates the pH value of the ARG/H_2_O impregnating solution.

A preliminary desorption test was performed by soaking ARG-5@SMSP in water (1 mg/mL) at 35 °C and under continuous stirring. Different batches were prepared by soaking 2 mg of loaded SMSP in 2 mL of water. At prefixed times (5 and 10 min), the solution of a single batch was completely collected, filtered through a syringe filter (4 mm × 0.45 µm) and the ARG content was analysed using 0.2% ninhydrin solution (procedure adapted from [31,32]) at 570 nm using a Lambda 25 Perkin Elmer spectrophotometer (Figure S3). The pH of the solution was measured before analysing ARG content and no modifications was observed due to the ARG desorption, due to the low concentration (91 µg/mL) of ARG in the final solution.

### 3.4. Instrumental Characterization

Textural properties of the samples were determined from Nitrogen adsorption–desorption isotherms obtained with a Micromeritics ASAP 2020 Plus Physisorption analyser (Micromeritics, Norcross, GA, USA). Before the measurement, the impregnated samples were outgassed at 70 °C for 2 h, while the unimpregnated SMSP were outgassed at 150 °C for 2 h. Specific surface area was calculated using the Barret–Emmett–Teller (BET) method in the relative pressure range of 0.119–0.196. Pore size distribution was obtained, using the Barlett–Joyner–Halenda (BJH) method, from the desorption branch of the isotherm. The total pore volume was determined at a relative pressure of about 0.93.

The morphology of the samples was characterized using a Field Emission Scanning Electron Microscope (FESEM Zeiss Merlin, Oxford Instruments, Abingdon-on-Thames, UK). The images were recorded on platinum metallized samples. Particle size distribution was calculated using the software “ImageJ.” (open source, https://imagej.net/, accessed on 1 October 2020) by analyzing 84 particles.

Powder X-Ray Diffraction (XRD) patterns were collected on Panalytical X’Pert PRO (Malvern Panalytical, Almelo, The Neatherlands) using Cu-Kα radiation (40 kV, 40 mA). The data were collected from 5° to 60° (2θ) with a step size of 0.026° (2θ). Low angle XRD patterns have been also collected from 1° to 10° (2θ) with a step size of 0.013° (2θ) (Figure S4). For unimpregnated SMSP, the peak due to the (100) reflection of the 2D hexagonal phase is observed, whereas the (110) and (200) reflections appear ill-defined [33]. The (100) reflection of the 2D hexagonal phase is not visible in the spectra of all samples containing ARG, because the presence of ARG molecules inside the mesopores is responsible for the reduction of the scattering contrast between the pores and the walls [34].

Fourier Transform Infrared (FT-IR) analysis was performed using an Equinox 55 spectrometer (Bruker, Billerica, MA, USA) on self-supported pellets. For ARG, the powder was diluted in KBr (Sigma-Aldrich). All samples were outgassed (residual pressure 0.1 Pa) at room temperature for 1 h. Spectra were obtained from 4000 cm^−1^ to 600 cm^−1^ with a resolution of 2 cm^−1^.

## 4. Conclusions

SMSP (with particle sizes ranging from 0.15 µm to 0.80 µm and average pore diameter of 2.4 nm) were synthesized, characterized, and loaded with ARG (with a final content of 9.1% *w*/*w*) through the wet impregnation technique by using water as a solvent. From nitrogen sorption analysis and FESEM analysis it could be inferred that the impregnation performed at the original basic pH of the ARG solution (i.e., pH 11) induced a significant modification of the porosity and of the surface of the particles, due to degradation ascribed to partial silica dissolution and reprecipitation. Impregnation performed adjusting the pH of the ARG solution to acidic (about 5) did not affect the carrier. The ARG was present in the SMSP carrier in its amorphous form in all samples. However, ARG molecules were present as zwitterionic species in the samples impregnated at basic pHs and as positive protonated species in the sample impregnated at acidic pH. The preliminary desorption test carried out in water revealed that the carrier impregnated at acidic pH (i.e., ARG-5@SMSP) was able to completely release ARG within 10 min, so confirming the possibility to use the SMSP carrier for arginine delivery.

## Figures and Tables

**Table 1:** SSA_BET_ and pore volume of SMSP and ARG-11@SMSP.

	SSA_BET_ (m^2^/g)	Pore Volume (cm^3^/g)
SMSP	1143	0.82
ARG-11@SMSP	786	0.51

## Data Availability

The data presented in this study are available in this article.

## Supplementary Materials

The following are available online at https://www.mdpi.com/article/10.3390/ijms222413403/s1.
