# Supplementary material for: The Role of the pH in the Impregnation of Spherical Mesoporous Silica Particles with L-Arginine Aqueous Solutions
Source: Int J Mol Sci. 2021 Dec 14;22(24):13403. doi: 10.3390/ijms222413403 (PMC8703690; doi:10.3390/ijms222413403)
Supplement: Supplementary file 1 [file ijms-22-13403-s001.zip › ijms-1491138-supplementary.pdf]

International Journal of *Molecular Sciences*

Supplementary Material

The Role of the pH in the Impregnation of Spherical Mesoporous Silica Particles with L-Arginine Aqueous Solutions

Sara Saber Younes Mohamed [1], Sonia Martinez [1], Mauro Banchero [1], Luigi Manna [1], Silvia Ronchetti [1] and Barbara Onida [1,*]

[1] Politecnico di Torino, Department of Applied Science and Technology (DISAT), Corso Duca degli Abruzzi, 24, 10129 Turin, Italy

* Correspondence: barbara.onida@polito.it

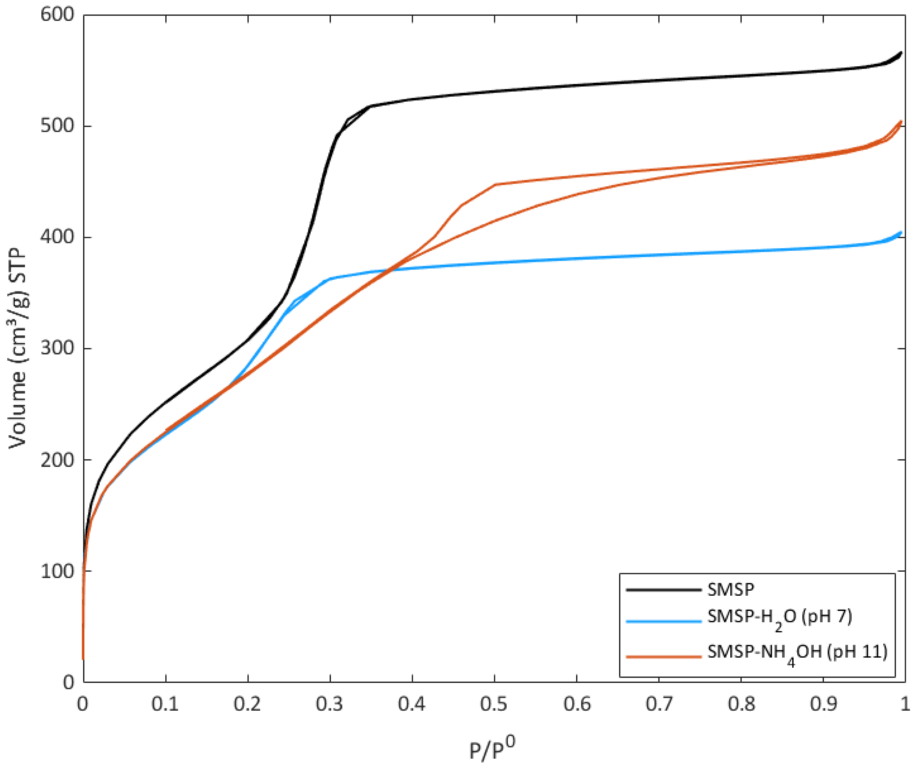

Figure S1. Nitrogen adsorption-desorption isotherms of SMSP, SMSP-H_2O and SMSP-NH_4OH.

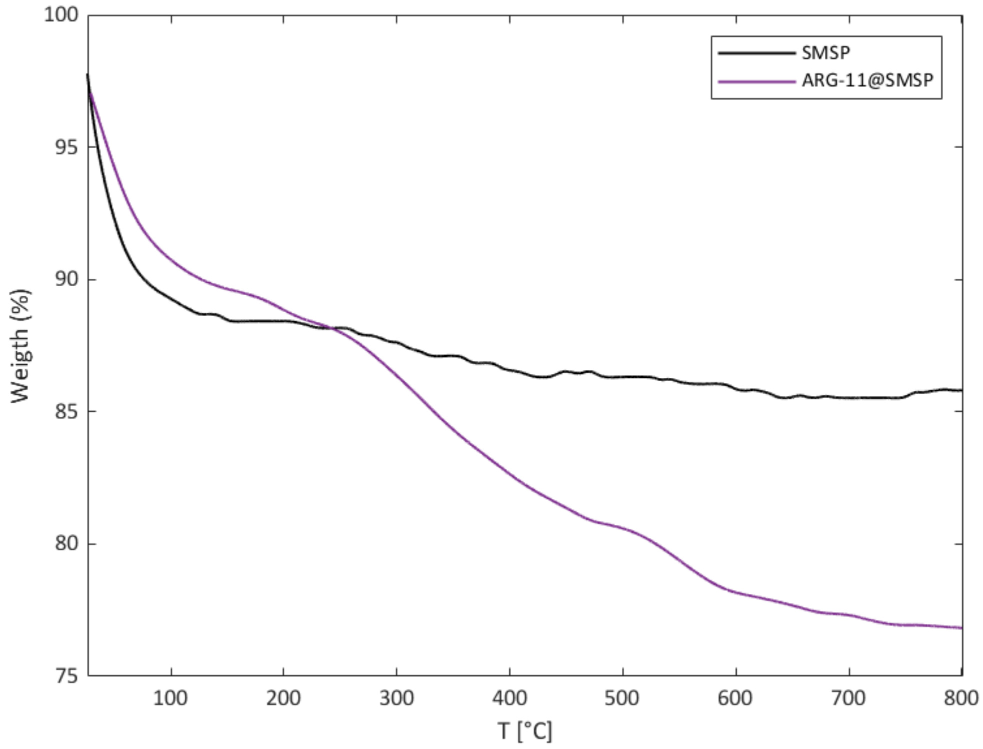

Figure S2. TGA curves of SMSP and ARG-11@SMSP (in air flow, heating rate 10 °C/min).

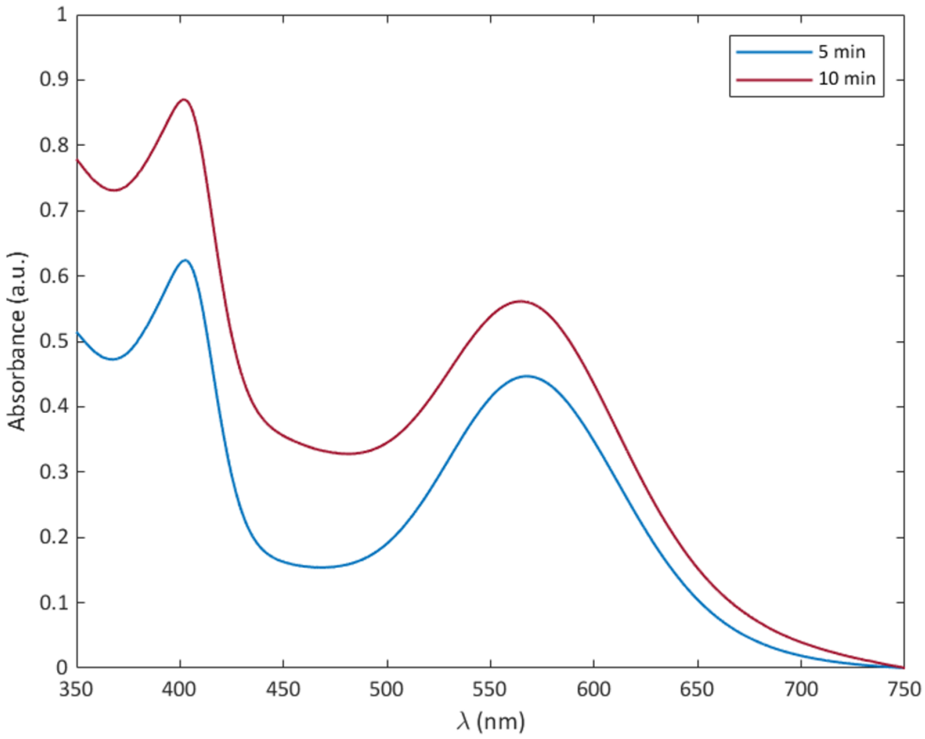

Figure S3. UV-VIS spectra of ARG solutions obtained after 5 and 10 minutes of soaking ARG-5@SMSP in water.

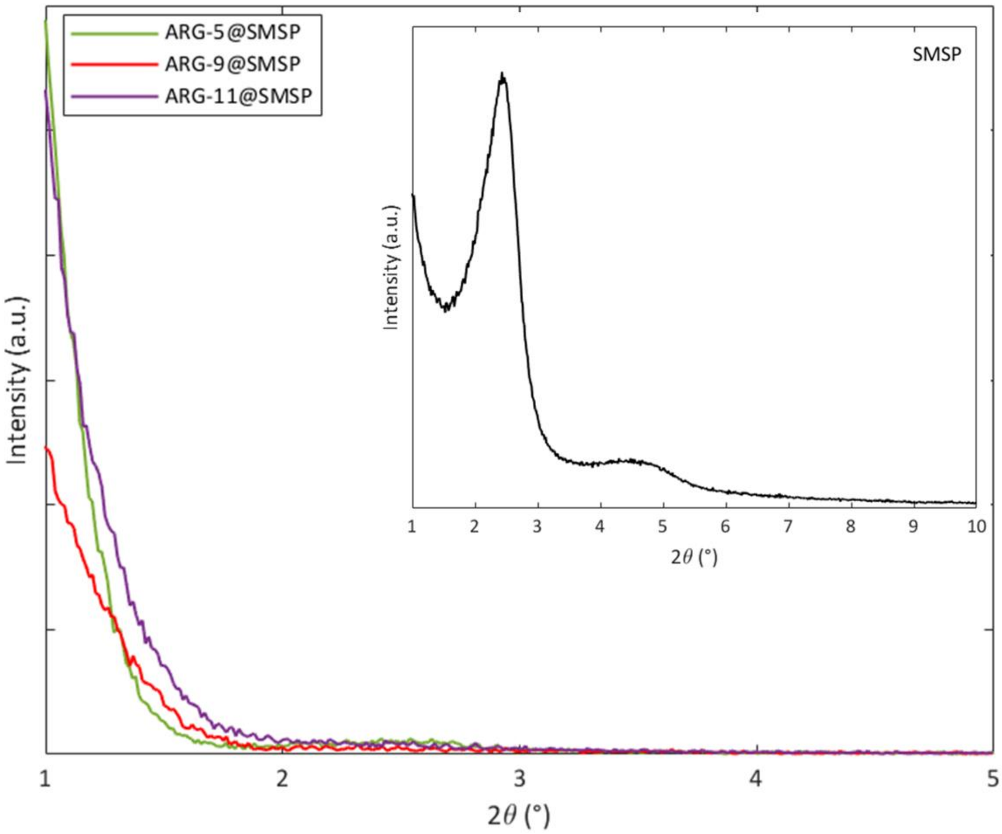

Figure S4. Low angle XRD patterns of SMSP (inset), ARG-5@SMSP, ARG-9@SMSP and ARG-11@SMSP.
